# Supplementary material for: Nitrogen Loss and Migration in Rice Fields under Different Water and Fertilizer Modes
Source: Plants (Basel). 2024 Feb 20;13(5):562. doi: 10.3390/plants13050562 (PMC10935088; doi:10.3390/plants13050562)
Supplement: Supplementary file 1 [file plants-13-00562-s001.zip › plants-2804640-Table S1.pdf]

Table S1. Water control criteria for different irrigation modes

**Irrigation and drainage mode**	**Water management metric**	**Regreen-ing stage**	**Earlier tillering stage**	**Later tillering stage**	**Jointing-booting stage**	**Heading-flowering stage**	**Milky ripening stage**	**Yellow ripening stage**
FSI	Lower limit of irrigation	10[1]	10	70 %θ_s[2]	10	10	10	Natural dry
	The upper limit of irrigation	30	60	0	70	60	40	
	The upper limit of rain storage	40	90	0	90	90	60	
II	Lower limit of irrigation	10	80 %θ_s	70 %θ_s	80 %θ_s	80 %θ_s	80 %θ_s	Natural dry
	The upper limit of irrigation	30	50	0	60	50	40	
	The upper limit of rain storage	40	80	0	80	80	60	

[1] The number unit in the table is "mm" indicates the field water depth, and [2] θ_s indicates the percentage of 20 cm soil saturated moisture. If the soil moisture content or the water depth reaches the lower limit of irrigation, fill the water to the upper limit of the irrigation, drain the water to the upper limit.
