# Supplementary material for: Nitrogen Loss and Migration in Rice Fields under Different Water and Fertilizer Modes
Source: Plants (Basel). 2024 Feb 20;13(5):562. doi: 10.3390/plants13050562 (PMC10935088; doi:10.3390/plants13050562)
Supplement: Supplementary file 1 [file plants-13-00562-s001.zip › plants-2804640-Table S2.pdf]

Table S2. Division of each birth period and fertilizer application amount

	Regreening			Early-tillering	Late-tillering	Jointing	Heading-flowering		Milky ripening	Yellow ripening
Date	6.27-7.6			7.7-8.6	8.7-8.10	8.11-8.23	8.24-9.16		9.17-10.10	10.11-10.23
Fertilizer [1]	P_2O_5	N	K_2O	N			N	K_2O		
NF	101.94	101.94	85.63	76.44			76.44	36.69		
C-80	81.55	81.55	68.50	61.15			61.15	29.35		

[1] The unit of fertilizer application is kg/hm[2].
