# Supplementary material for: Nitrogen Loss and Migration in Rice Fields under Different Water and Fertilizer Modes
Source: Plants (Basel). 2024 Feb 20;13(5):562. doi: 10.3390/plants13050562 (PMC10935088; doi:10.3390/plants13050562)
Supplement: Supplementary file 1 [file plants-13-00562-s001.zip › plants-2804640-Table S3.pdf]

Table S3. Different treatment and irrigation displacement

Treatment	FW	FA	FA-80	IW	IA	IA-80
Irrigation of water	1387.43[1] ±73.46a	1332.23 ±21.85ab	1281.52 ±11.51b	1182.02 ±33.43c	1158.69 ±1.88cd	1103.30 ±35.12d
Surface water drainage	0	0	0	0	0	0
Groundwater drainage	250.33±4.87a	259.77±8.88a	247.44 ±14.19a	164.44 ±15.55b	163.15±7.70b	158.44±8.88b

[1] The unit of fertilizer application is mm. The letters in the table indicate the significant difference between the treatments ($P < 0.05$).
