# Supplementary material for: Nitrogen Loss and Migration in Rice Fields under Different Water and Fertilizer Modes
Source: Plants (Basel). 2024 Feb 20;13(5):562. doi: 10.3390/plants13050562 (PMC10935088; doi:10.3390/plants13050562)
Supplement: Supplementary file 1 [file plants-13-00562-s001.zip › plants-2804640-Table S4.pdf]

Table S4. Loss load of nitrogen

Nitrogen form	Treat-ment	Loss load of nitrogen					Amount
		Regreening	Tillering	Jointing-booting	Hrading-flowering	Milky-riping	
TN	FW	0.248±0.086b[1]	0.979±0.158b	0.553±0.122a	0.418±0.111b	0.501±0.122a	2.699±0.238a
	FA	0.288±0.063a	1.105±0.233a	0.370±0.026b	0.484±0.068a	0.451±0.087b	2.698±0.212a
	FA-80	0.251±0.090b	0.849±0.098c	0.350±0.055b	0.483±0.088a	0.446±0.058b	2.379±0.182b
	IW	0.243±0.063b	0.854±0.125c	0.354±0.086b	0.404±0.075b	0.274±0.045c	2.129±0.155c
	IA	0.249±0.026b	0.437±0.056d	0.334±0.065b	0.247±0.046e	0.229±0.015c	1.496±0.122e
	IA-80	0.192±0.050c	0.459±0.056d	0.366±0.055b	0.290±0.058c	0.270±0.058c	1.577±0.155d
NH_4^+-N	FW	0.207±0.065b	0.894±0.202a	0.442±0.086a	0.390±0.089c	0.468±0.105a	2.401±0.122a
	FA	0.257±0.078a	0.930±0.158a	0.343±0.086b	0.468±0.068b	0.379±0.062ab	2.377±0.152a
	FA-80	0.214±0.065b	0.671±0.102c	0.326±0.080b	0.504±0.112a	0.321±0.069b	2.036±0.088b
	IW	0.205±0.058b	0.810±0.185b	0.329±0.102b	0.388±0.065c	0.226±0.068d	1.958±0.102c
	IA	0.181±0.045c	0.355±0.087d	0.327±0.030b	0.188±0.026e	0.204±0.022d	1.255±0.158e
	IA-80	0.166±0.026c	0.389±0.020d	0.320±0.056b	0.264±0.058d	0.219±0.090d	1.358±0.088d
NO_3^--N	FW	0.018±0.002c	0.056±0.020c	0.054±0.012b	0.054±0.021b	0.049±0.012a	0.231±0.026b
	FA	0.029±0.010b	0.075±0.026b	0.077±0.021a	0.069±0.021a	0.054±0.012a	0.304±0.030a
	FA-80	0.051±0.015a	0.086±0.026a	0.045±0.012c	0.062±0.021a	0.056±0.010a	0.300±0.025a
	IW	0.056±0.022a	0.068±0.021bc	0.045±0.012c	0.026±0.006c	0.027±0.005b	0.222±0.030b
	IA	0.062±0.022a	0.048±0.010d	0.028±0.010d	0.028±0.010c	0.033±0.010b	0.199±0.018c
	IA-80	0.030±0.005b	0.062±0.026bc	0.034±0.012d	0.025±0.010c	0.036±0.012b	0.187±0.020c

[1] The letters in the table indicate the significant difference in different period of rice between the treatments ($P < 0.05$).
