# Supplementary material for: Nitrogen Loss and Migration in Rice Fields under Different Water and Fertilizer Modes
Source: Plants (Basel). 2024 Feb 20;13(5):562. doi: 10.3390/plants13050562 (PMC10935088; doi:10.3390/plants13050562)
Supplement: Supplementary file 1 [file plants-13-00562-s001.zip › plants-2804640-Table S5.pdf]

Table S5 Change of nitrogen concentration in soil vertical profile

Nitrogen form	Deep	FW	FA	FA-80	IW	IA	IA-80
TN	-18	0.637±0.125c[1]	0.799±0.126b	0.790±0.118b	0.633±0.142c	0.835±0.092a	0.807±0.188b
	-36	0.618±0.085c	0.688±0.102b	0.678±0.055b	0.623±0.125c	0.718±0.118a	0.701±0.132ab
	-56	0.629±0.098a	0.526±0.082c	0.533±0.062bc	0.613±0.075a	0.549±0.062b	0.559±0.080b
NH_4^+-N	-18	0.395±0.083c	0.444±0.103b	0.467±0.112a	0.388±0.088c	0.471±0.088a	0.458±0.068ab
	-36	0.384±0.026a	0.392±0.070a	0.407±0.088a	0.387±0.036a	0.400±0.076a	0.389±0.085a
	-56	0.402±0.045a	0.346±0.026b	0.353±0.050b	0.393±0.045a	0.330±0.042c	0.322±0.040c
NO_3^--N	-18	0.171±0.021d	0.351±0.030b	0.367±0.056b	0.206±0.069c	0.403±0.062a	0.380±0.099a
	-36	0.169±0.056d	0.262±0.041a	0.254±0.026a	0.203±0.036c	0.268±0.026a	0.244±0.026b
	-56	0.164±0.042c	0.151±0.026c	0.163±0.035c	0.199±0.012b	0.218±0.035a	0.210±0.048a

[1] The letters in the table indicate the significant difference in different nitrogen form between the treatments ($P < 0.05$).
