# Supplementary material for: Nitrogen Loss and Migration in Rice Fields under Different Water and Fertilizer Modes
Source: Plants (Basel). 2024 Feb 20;13(5):562. doi: 10.3390/plants13050562 (PMC10935088; doi:10.3390/plants13050562)
Supplement: Supplementary file 1 [file plants-13-00562-s001.zip › plants-2804640-Table S6.pdf]

Table S6 Precipitation distribution of rice at each growth stage

	**Regreening**	**Tillering**	**Jointing-booting**	**Hrading-flowering**	**Milky-riping**	**Amount**
Precipitation（mm）	2.80	50.40	0.00	30.70	53.40	137.30
Average daily precipitation（mm/d）	0.28	1.44	0.00	1.28	2.23	1.20
Precipitation proportion（%）	2.04	36.71	0.00	22.36	38.89	/
