# Supplementary figures and images for: Nitrogen Loss and Migration in Rice Fields under Different Water and Fertilizer Modes
Source: Plants (Basel). 2024 Feb 20;13(5):562. doi: 10.3390/plants13050562 (PMC10935088; doi:10.3390/plants13050562)

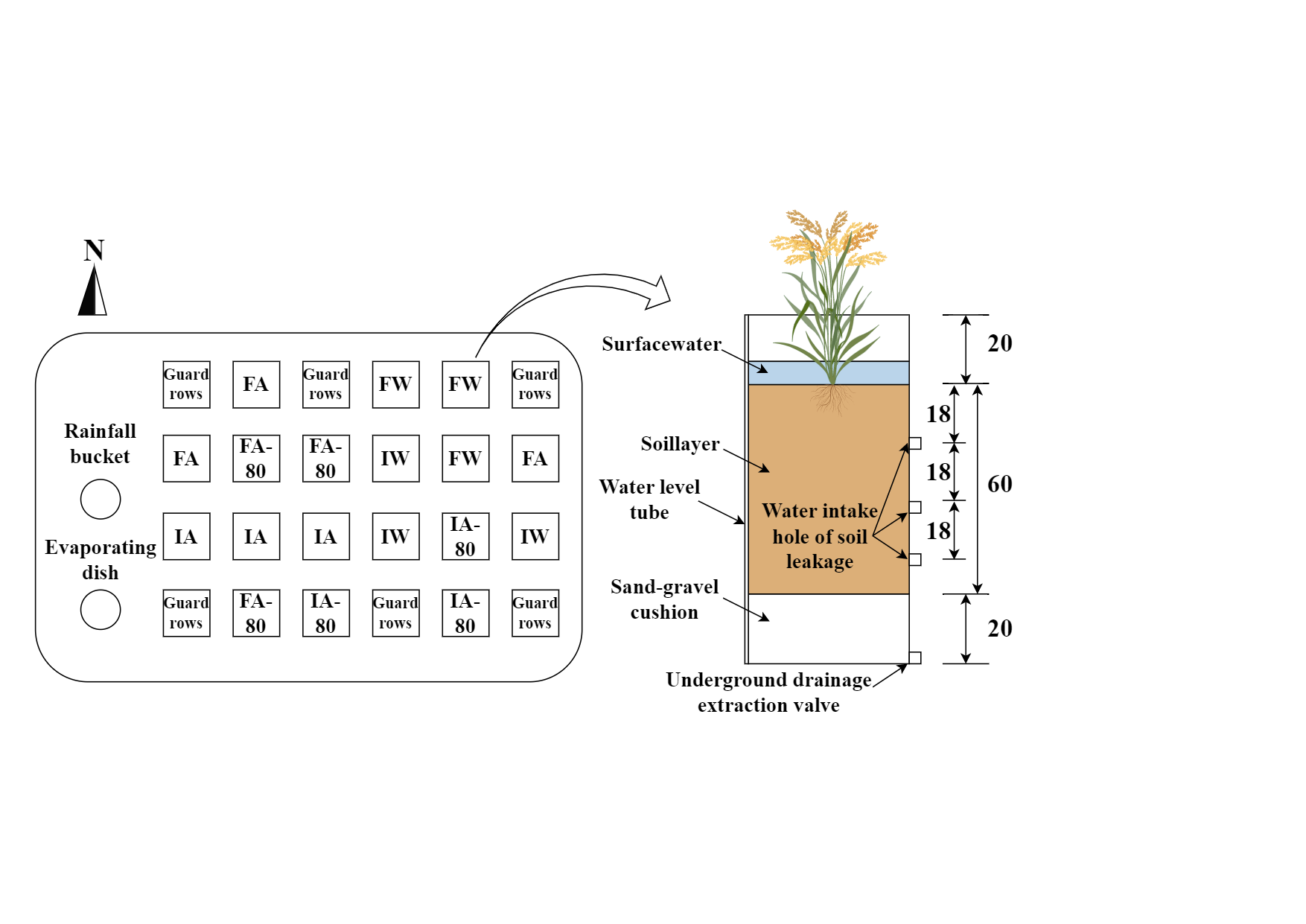

Supplement: Supplementary file 1 [file plants-13-00562-s001.zip › plants-2804640-Figure S1.jpg]

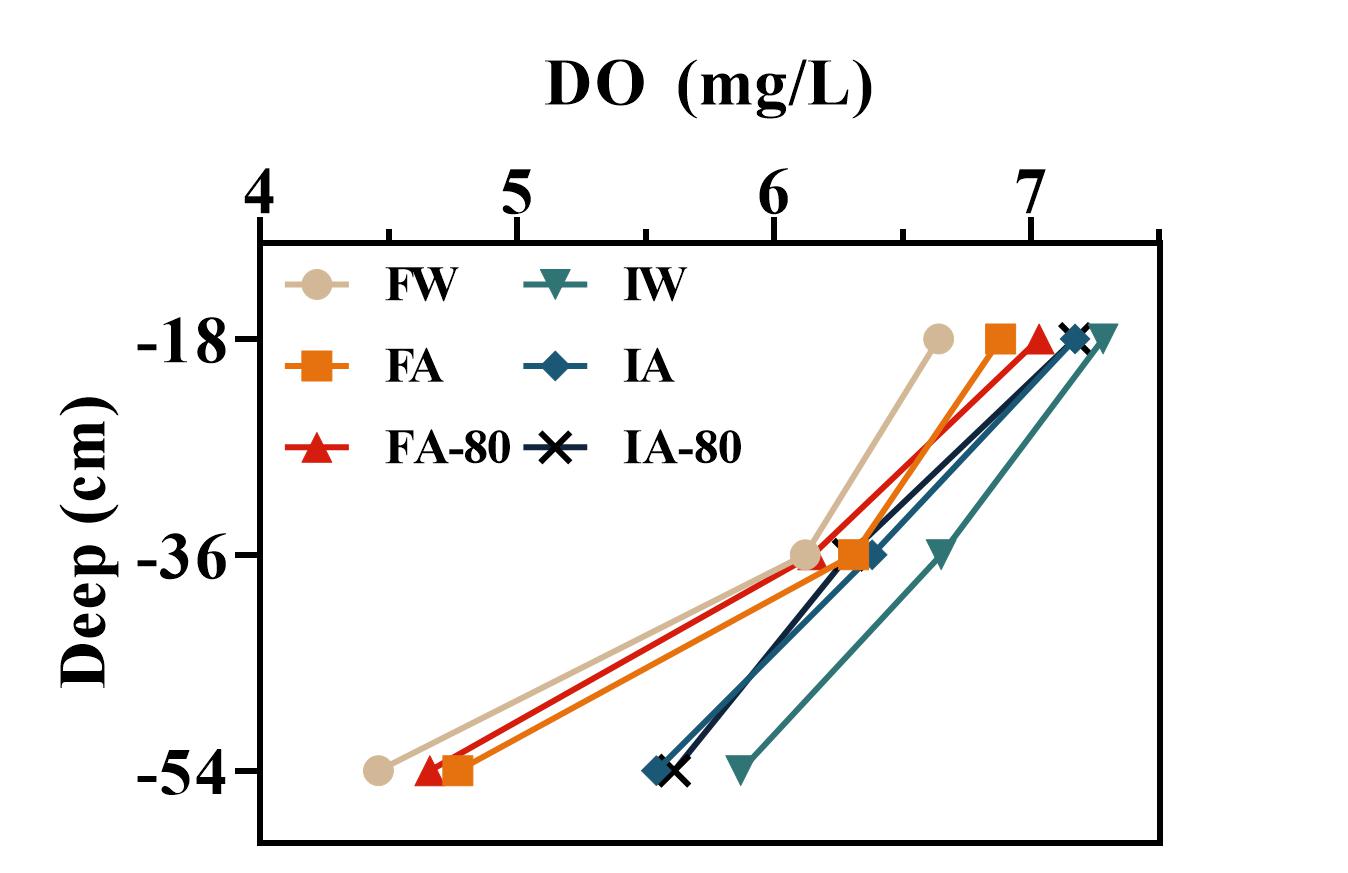

Supplement: Supplementary file 1 [file plants-13-00562-s001.zip › plants-2804640-Figure S2.jpg]
